# Massive gastric dilation caused by gastric outlet obstruction in the setting of peptic ulcer disease—A case report

**DOI:** 10.1016/j.ijscr.2020.04.015

**Published:** 2020-05-08

**Authors:** C.S. Costa, N. Pratas, H. Capote

**Affiliations:** Department of General Surgery, North Alentejo Local Unit E.P.E., Portugal

**Keywords:** Gastric outlet obstruction, Peptic ulcer disease, Case report

## Abstract

**Background:**

The prevalence of Peptic Ulcer Disease has decreased as did its elective surgical treatment, however its complications continue to occur.

Gastric Outlet Obstruction is the least frequent complication of Peptic Ulcer disease.

**Case summary:**

In this report, we present a case of massive gastric dilation due to gastric outlet obstruction, that needed emergency surgery due to perforation. A subtotal gastrectomy with a Billroth II reconstruction was performed.

**Discussion:**

Usually non-operative management is tried first, with medical therapy and endoscopic dilation. Emergent surgery is rarely needed, but in this patient, despite trying to optimize his condition first, the ulcer perforation precipitated surgical management.

**Conclusion:**

Although Gastric Outlet Obstruction is the least frequent complication of Peptic Ulcer Disease and usually non-operative treatment is tried first, surgery may be necessary.

## Highlights

• Gastric Outlet Obstruction is the least frequent complication of Peptic Ulcer disease.
• We present a case of massive gastric dilation due to gastric outlet obstruction, that needed emergency surgery due to perforation.
• We present a case of massive gastric dilation due to gastric outlet obstruction, that needed emergency surgery.

## 1 Introduction

Peptic Ulcer Disease (PUD) is a common gastro-duodenal disorder, affecting as many as 4% of the population [1,2]. Due to efficient eradication of *Helicobacter Pylori* (HP) and use of protein pump inhibitors (PPI’s), the prevalence of PUD has decreased as did its elective surgical treatment [[1], [2], [3], [4]]. However complications of PUD continue to occur [5].

The classical complications of PUD are bleeding, perforation and gastric outlet obstruction (GOO) [[6], [7], [8], [9]]. GOO is the least frequent complication of PUD in developed countries and it is rarely an emergency [3], whereas bleeding and perforation are [4]. In this context, GOO in the need for surgery is usually a result of chronic inflammation and scarring due to ongoing PUD [2,10]. The cardinal symptoms are anorexia, nausea, vomiting and epigastric pain [7,10,11]. Weight loss and malnutrition are often present [10]. The stomach can become massively dilated and lose its muscular tone rapidly [10].

Treatment for GOO may be non- operative, using medical therapy and endoscopic pneumatic dilation, or operative [5,9]. There are a few surgical procedures possible, including gastrectomy, subtotal gastrectomy with a Billroth II reconstruction, vagotomy combined with antrectomy or vagotomy combined with a drainage procedure [5,12].

In this article, we present a case of massive gastric dilation due to benign GOO.

This paper was reported in line with the SCARE criteria [13].

## 2 Timeline

Day 1 – Emergency Room visit for abdominal pain: CT showed GOO

Day 3 – Central venous catheter placed. Iatrogenic pneumothorax; drainage with chest tube

Day 4 – Ulcer perforation. Emergency Laparotomy: Subtotal Gastrectomy with Billroth II reconstruction

Day 27 – ICU discharge

Day 29 – Chest Tube removal Day 43 – Hospital Discharge

## 3 Case

A 54 year old male, smoker, with no prior illnesses or surgeries, presented to the emergency room with sudden epigastric abdominal pain. He denied nausea or vomiting but on further inquiry he revealed that he had anorexia and vomited occasionally in the last 7 months, having lost 7 kg in that time span. He looked malnourished and his abdomen was distended and generally tender. A computed tomography (CT) was performed revealing massive gastric dilation due to pyloric stenosis. The patient was initially managed with nasogastric drainage, intravenous (iv) fluids, iv PPI and placement of a right subclavian central line for parenteral feeding. During placement of this central line an iatrogenic pneumothorax occurred and right chest tube was placed.

On day 3 the patient’s abdominal pain increased significantly and he showed signs of peritonitis. Another CT was ordered and a pneumoperitoneum was evident. The patient was rushed to the Operating Room and an exploratory laparotomy was performed, revealing chemical peritonitis, a 1 cm perforation on a pre- pyloric ulcer with pyloric scarring and stenosis. A subtotal gastrectomy was performed with a Billroth II reconstruction.

Post-operatively the patient stayed in the Intensive Care Unit (ICU) for 23 days. He had septic shock due to an infected jugular central line that was placed later, and needed antibiotics and aminergic support.

He also needed the chest drainage (which was changed several times) for 26 days until complete resolution of the pneumothorax, However, once extubated, he had no trouble in resuming oral feeding. At day 34 the patient exhibited signs of shock again, and the ordered CT showed a sub-phrenic collection that resolved with an 8 day course of Meropenem and Vancomycin. He was finally discharged 40 days after surgery. The pathology of the surgical specimen confirmed benign gastric ulcer with no dysplasia associated.

**Figs. 1 and 2:**
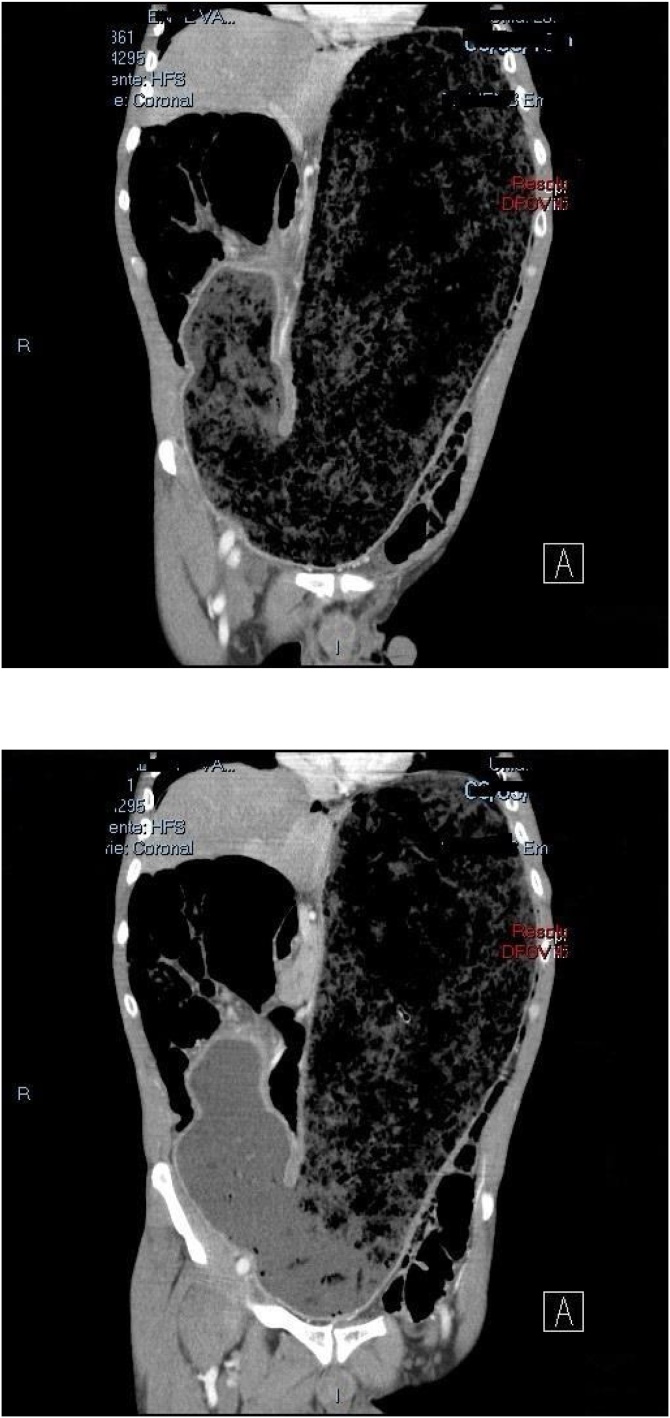
CT of massive gastric dilation due to pyloric stenosis (2018).

A month after discharge he was seen in the outpatient clinic and was well, tolerating diet and had gained weight. The patient was not tested for HP status and has been called for new consultation to determine if he is indeed positive for infection so that eradication can be performed if needed (Figs. 1 and 2, Figs. 1 and 2).

## 4 Discussion

Patients presenting with GOO, should be optimized initially with nasogastricaspiration, fluid resuscitation, PPI’s and parenteral nutrition [5]. Attention should be made to place a large bore nasogastric tube as the stomach may have large food fragments that clog the tube. In the past PUD was the main cause of GOO, but nowadays malignant obstruction is more frequent, and should be ruled out [7,10,11,14].

**Fig. 3:**
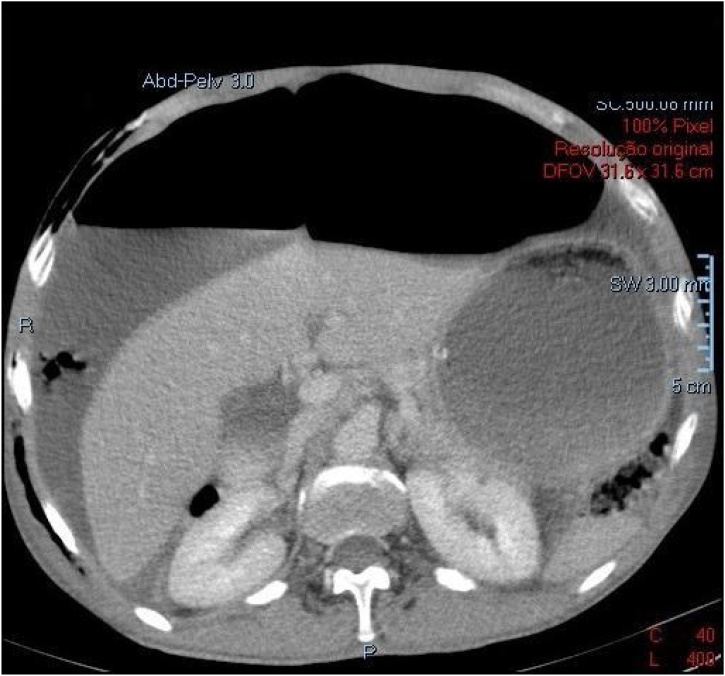
CT of Pneumoperitoneum after perforation (2018).

Usuallynon-operative management is tried first, with medical therapy and endoscopic dilation [5,9]. Emergent surgery is rarely needed [3], but in this patient, despite trying to optimize his condition first, the ulcer perforation precipitated surgical management. The choice of procedure was easy because performing a subtotal gastrectomy, allowed removal of the ulcer, the scarred pylorus and proximal duodenum and of a large part of the stomach that was occupying the peritoneal cavity. The Billroth II anastomosis was easy and quick to perform, which was a good choice in the setting of emergency surgery. Despite the patient’s long hospital stay we managed to obtain a favorable final result, solving his GOO and giving him quality of life (Fig. 3).

## 5 Conclusion

Although GOO is the least frequent complication of PUD and usually non-operative treatment is tried first, it is important to have an understanding of its surgical management, since surgery may be necessary.

## Declaration of Competing Interest

All authors declare no conflict of interest.

## Sources of funding

None.

## Ethical approval

For case reports, no official approval of the ethics committee is required. The patient gave informed consent for writing the case report.

## Consent

Informed consent was obtained from the patient for publication of this case report and accompanying images.

## Authors contribution

Both C.S. Costa and N. Pratas were involved in patient care and wrote the manuscript.

H. Capote was involved in patient care and contributed to writing the manuscript.

All authors approve the last version of the manuscript.

## Registration of research studies

Not Applicable.

## Guarantor

H. Capote.

## Provenance and peer review

Not commissioned, externally peer-reviewed.
